# Effectiveness of telerehabilitation intervention to improve pain and physical function in people with patellofemoral pain syndrome: study protocol for a randomized controlled trial

**DOI:** 10.1186/s13063-024-08047-3

**Published:** 2024-03-19

**Authors:** Negar Amirabadi, Masumeh Hessam, Saeideh Monjezi, Farshad Molhemi, Mohammad Mehravar, Pardis Hosseinpour

**Affiliations:** 1 Student Research Committee, Ahvaz Jundishapur University of Medical Sciences, Ahvaz, Iran; 2 Rehabilitation Research Center, Ahvaz Jundishapur University of Medical Sciences, Ahvaz, Iran; 3 Department of Physiotherapy, School of Rehabilitation Sciences, Ahvaz Jundishapur University of Medical Sciences, Ahvaz, Iran

**Keywords:** Randomized controlled trial, Telerehabilitation, Patellofemoral pain syndrome, Smartphone application, Exercise therapy, Face-to-face physical therapy

## Abstract

**Background:**

Patellofemoral pain syndrome (PFPS) is a common musculoskeletal condition in young and active adults. Exercise therapy is an essential part of rehabilitation in people with PFPS (PwPFPS). Telerehabilitation is an innovative treatment approach that has been used in several musculoskeletal conditions. This study aims to investigate the non-inferiority of telerehabilitation through a smartphone application, the *Vito App*, compared to face-to-face physical therapy on reducing pain and improving physical function, quality of life, and psychological factors.

**Methods:**

This randomized controlled trial will include 60 PwPFPS. to a control group (face-to-face physical therapy) or an experimental group (telerehabilitation). The intervention for both groups consists of stretching, strengthening, balance, and functional exercises for 6 weeks and three sessions per week. The primary outcomes are pain intensity by visual analog scale (VAS), physical function by the Kujala questionnaire and functional tests including the bilateral squat, anteromedial lunge, and step down, and quality of life by the Knee Injury and Osteoarthritis Outcome Score (KOOS) questionnaire quality of life subscale. Secondary outcomes are psychological factors such as anxiety and depression assessed with the Hospital Anxiety and Depression Scale (HADS) questionnaire, kinesiophobia assessed with the Tampa scale, and pain catastrophizing assessed with the Pain Catastrophizing Scale (PCS). Assessments will be held in 3 phases: pre-test (before the intervention), post-test (after the 6 weeks of intervention), and follow-up (1 month after the end of the intervention).

**Discussion:**

We expect that both the control group and experimental group will show similar improvements in clinical and psychological outcome measures. If our hypothesis becomes true, PwPFPS can use telerehabilitation as a practical treatment approach. Telerehabilitation can also enhance accessibility to rehabilitation services for active adults and for people living in remote and rural areas.

**Trial registration:**

Iranian Registry of Clinical Trials (IRCT) IRCT20201112049361N1. Registered on 29 October 2022.

**Supplementary Information:**

The online version contains supplementary material available at 10.1186/s13063-024-08047-3.

## Background

Patellofemoral pain syndrome (PFPS) is a common musculoskeletal problem characterized by anterior and peripatellar knee pain [1]. Activities such as running, jumping, stair climbing and descending, prolonged sitting, squatting, and kneeling are the main contributors to pain in PFPS [1, 2]. This condition is common in the general population, as well as among physically active adults, runners, and military personnel [1, 3]. Females are likely to experience patellofemoral pain twice as much as men [3, 4]. Almost 70–90% of people with PFPS (PwPFPS) experience recurrent or chronic knee pain in the following years of their lives [1]. The PFPS in younger individuals may develop patellofemoral osteoarthritis later in life [1, 5].

Biomechanical impairments are associated with pain and activity limitations in PwPFPS [6–8]. According to a recent systematic review, PwPFPS have significant strength deficits in hip abduction, flexion, extension, and external rotation [9]. Maclachlan et al. also reported that psychological factors such as anxiety, depression, kinesiophobia, and pain catastrophizing may correlate with pain and reduced physical function among this population [10].

Physical therapy is one of the most effective interventions to reduce pain and improve physical function in PwPFPS. Among variable physical therapy interventions, therapeutic exercises can significantly reduce pain, improve physical ability, and enhance long-term recovery in PwPFPS [11–14].

Telerehabilitation refers to the use of technologies to provide and deliver rehabilitation services via telemedicine methods [15]. Telerehabilitation can be cost-effective, increase the adherence of patients to the rehabilitation process, and patients can have an influential role in the management of their condition [15–18]. In addition, it can improve the continuity of treatment as long as it allows the patients to maintain contact with their therapists for the long term after the end of the sessions [15, 18, 19]. Telerehabilitation is a more effective and convenient option than face-to-face physical therapy for patients with physical disabilities who have difficulties with traveling and who live in remote areas [15]. A systematic review by Kairy et al. shows that telerehabilitation can lead to similar clinical outcomes compared with face-to-face physical therapy, and both physical therapists and patients accept it as an effective treatment [20].

Mobile health (mHealth) is a form of telerehabilitation that involves the delivery of healthcare services through smartphones [21]. Smartphone applications can provide real-time communication between patients and therapists, collect data and information from patients, and provide quick access to the collected data for the physical therapist, thereby improving the quality of healthcare services delivery [21, 22].

Previous studies indicated that telerehabilitation has positive outcomes similar to face-to-face physical therapy in patients with neurological [23, 24], musculoskeletal [25, 26], and cardiopulmonary disorders [27]. A systematic review indicated that in patients with musculoskeletal disorders, telerehabilitation can be used as an alternative to face-to-face physical therapy [28]. In addition, telerehabilitation had positive effects on pain, disability, and quality of life in people with low back pain and knee osteoarthritis [29–32]. Until now, a few studies have investigated the effectiveness of telerehabilitation in PwPFPS. In a prospective clinical trial, Albornoz-Cabello et al. reported that a tele-prescription program through a pamphlet containing the description of exercises and phone call control by a physical therapist could be effective in reducing pain and disability in PwPFPS during the COVID-19 pandemic [33]. In a study by Arslan et al., telerehabilitation through online supervised exercise programs decreased pain and kinesiophobia and increased the quality of life in female PwPFPS [34]. This study compared the effect of an online supervised exercise program and a home exercise program with a control group that did not receive any intervention. Therefore, in this study, the effect of telerehabilitation was not compared with face-to-face or supervised rehabilitation programs. In 2023, Lee et al. indicated that telerehabilitation is as effective as supervised rehabilitation in improving psychological and functional outcomes in women with PFPS [35]. This study only included female PFPS patients, used a quasi-experimental design with no randomization, and had no follow-up to assess the long-term effectiveness of telerehabilitation. According to the mentioned shortcomings of the previous studies, we aimed to examine the effectiveness of telerehabilitation using a smartphone application will be examined in this study and compared with face-to-face physical therapy in the short-term and mid-term for PwPFPS.

## Methods

### Study aims

The primary objective of this randomized controlled trial is to determine whether exercise therapy via telerehabilitation and mHealth is as effective as the same face-to-face physical therapy program in reducing pain and improving physical function and quality of life in PwPFPS.

The secondary objectives of this study are to evaluate the effectiveness of telerehabilitation in improving psychological factors, including anxiety, depression, kinesiophobia, and pain catastrophizing, and compare them to face-to-face physical therapy. We will also investigate the adherence of participants to the treatment plan. We hypothesize that telerehabilitation and face-to-face physical therapy will both be effective in pain reduction, and improving physical function, quality of life, and psychological factors; these two intervention methods have no superiority over each other.

### Study design, randomization, and allocation

The present protocol is a non-inferiority, single-blinded, randomized controlled trial with two parallel groups (face-to-face physical therapy and telerehabilitation groups) and a 1:1 allocation ratio. The ethics committee of AJUMS (IR.AJUMS.REC. 1401.282) approved the protocol and it is registered (no: IRCT20201112049361N1.) in the Iranian Registry of Clinical Trials (IRCT). This study protocol follows the SPIRIT (Standard Protocol Items: Recommendations for Interventional Trials) checklist which is available as an additional file (Additional file 1) [36].

**Fig. 1:**
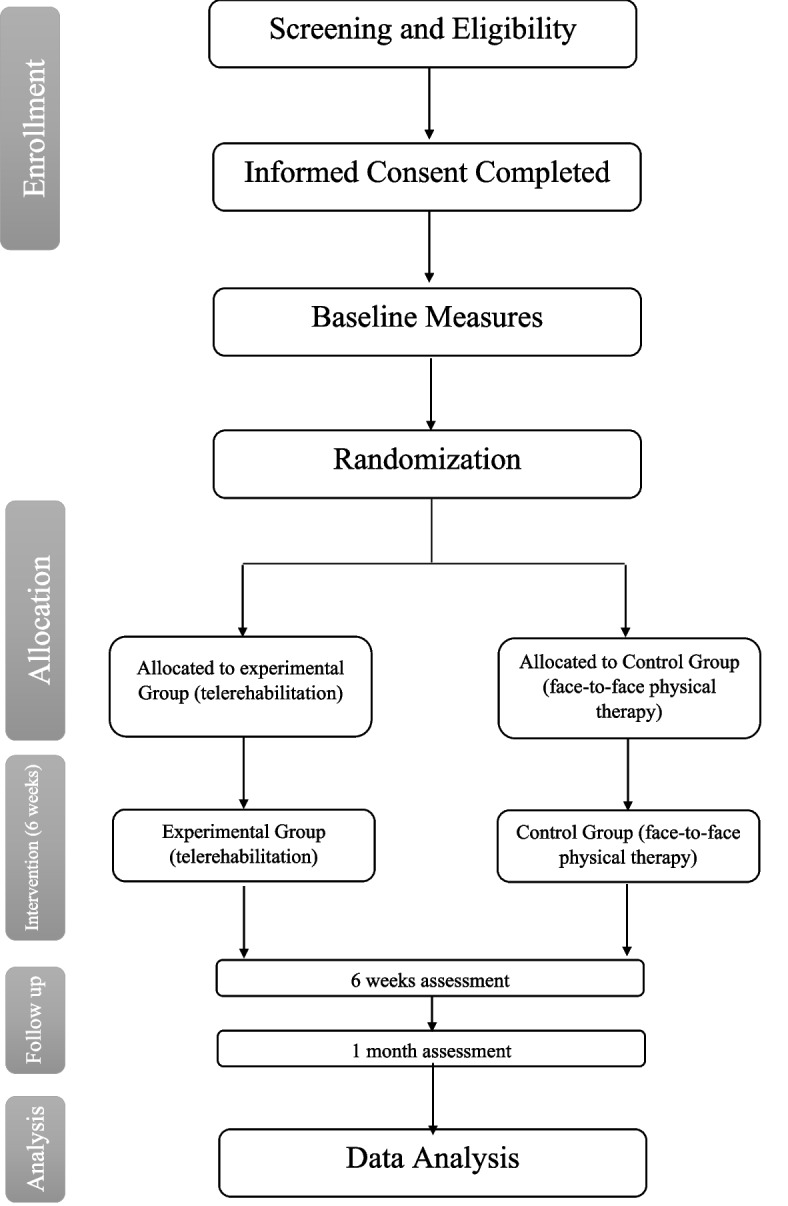
Flow chart of the study

Participants will be randomly assigned to either the control group (face-to-face physical therapy) or the experimental group (telerehabilitation) using the stratified permuted block method (Fig. 1). Participants will be randomly assigned to groups based on age, sex, and level of activity as determined by the Tegner Activity Scale to ensure similarity of these variables. A computerized random allocation sequence with different block sizes (4 and 6) will be provided by an independent investigator who is not a member of the research team. An uninvolved party in the assessment will open the sealed envelopes.

### Study setting and participants

The study will take place at the Rehabilitation Research Center of the Ahvaz Jundishapur University of Medical Science (AJUMS) and the physical therapy clinic of the rehabilitation department of AJUMS. The target population in this study will be PwPFPS. Participants will be recruited through advertisements on social media and at outpatient physical therapy centers in Ahvaz, Iran. The trial coordinator (N.A.) will interview participants on the phone to verify whether they meet the inclusion criteria. Eligible participants will be invited to a face-to-face evaluation. After explaining the purpose of the study, written informed consent will be obtained from all the participants. An independent person, who is a member of the data monitoring committee of the Ahvaz Jundishapur University of Medical Science (AJUMS), will monitor the data collection process.

### Inclusion criteria

The inclusion criteria are as follows: (1) age between 18 and 55; (2) access to the internet and mobile phone; (3) pain around, behind, and in front of the patella that is caused by ascending and descending of the stairs, knee flexion, squatting, and prolonged sitting; (4) knee pain for at least 1 month; and (5) visual analog scale (VAS) rating of pain during activity more than three.

### Exclusion criteria

The exclusion criteria are (1) knee meniscus, ligament, or tendon injuries; (2) patellar dislocation, subluxation, or fractures; (3) conservative or surgical treatment of the affected knee in less than the previous 6 months; and (4) neuromuscular, metabolic, and rheumatologic disorders.

### Intervention

The intervention procedure lasts for 6 weeks with three sessions per week (18 sessions and each session lasts half an hour). Participants will perform a set of therapeutic exercises including stretching, strengthening, balance, and functional exercises. The therapeutic exercises and their progress during 6 weeks in the experimental and control groups are based on the American Physical Therapy Association (APTA) Clinical Practice Guidelines for PFPS in 2021 [13]. The therapeutic exercises are precisely the same in both groups. The only difference between the experimental and control groups is the method of therapeutic exercise delivery, which is face-to-face in the control group and via a smartphone application in the experimental group. The training program includes three stages. Every stage consists of the same stretching exercises at the beginning of the session. The stretching exercises target the hamstring, quadriceps, iliotibial band, and gastro-soleus muscles. The first stage includes the first 2 weeks. It consists of strengthening exercises such as sitting knee extension, quadriceps setting, squatting (0 to 45° knee angle), side lying hip abduction, prone hip external rotation, and standing hip abduction. The second stage, which is the second 2 weeks (weeks 3 and 4), consists of strengthening and balance exercises such as prone hip extension, squatting (45 to 60° knee angle), single leg stance, single leg heel raise, lunge (45° knee angle), and seated hip external rotation. Participants will perform the exercises in the first two stages in 3 sets with ten repetitions. The third stage (weeks 5 and 6) focuses on functional exercises in addition to strengthening and balance exercises such as squatting (90° knee angle), single leg squat, lateral step up, front step up, and lunge (90° knee angle). Participants will perform explained exercises in the final stage in 3 sets with fifteen repetitions (Table 1).

**Table 1:** Exercise plan

Weeks	Target	Exercises	Sets/Repetitions
1–2	• Improve muscle strength	1. Sitting knee extension 2. Quadriceps setting 3. Squatting (0 to 45° knee angle) 4. Side lying hip abduction 5. Prone hip external rotation 6. Standing hip abduction	3 sets/10 repetitions
3–4	• Improve muscle strength • Improve balance	1. Prone hip extension 2. Squatting (45 to 90° knee angle) 3. Single leg stance 4. Single leg heel raise 5. Lunge (45° knee angle) 6. Seated hip external rotation	3 sets/10 repetitions
5–6	• Improve muscle strength with more force • Improve balance • Improve knee function	1. Squatting (90° knee angle) 2. Single leg squat 3. Lateral step up 4. Front step up 5. Lunge (90° knee angle)	3 sets/15 repetitions

The physical therapist for both groups, one of the research team members (N.A.), will be blinded to the participant’s baseline assessments. The assigned physical therapist is trained and experienced in training patients with musculoskeletal disorders. At the end of each session, the participants will be asked to report their pain intensity using the VAS. In case of increased pain or difficulties during exercises, the physical therapist will modify the sets and repetitions of each exercise for the next session. Any concomitant knee-related physical therapy intervention and corticosteroid consumption during the treatment procedure in this study is prohibited for participants.

### Face-to-face physical therapy program

The control group will perform the exercises in the physical therapy clinic at the rehabilitation department of AJUMS under the supervision of the physical therapist. The physical therapist will explain the exercises to the participants, supervise their progress, and ensure that they adhere to the exercises.

### Telerehabilitation program

The telerehabilitation group will perform the same exercises as the control group using a mHealth application named the *Vito App*. During the first evaluation session, the assigned physical therapist will install the application on the participant’s smartphone, provide instructions on how to use it, and explain its various features.

In this group, the physical therapist will provide weekly phone calls to participants and monitor their progress through the application calendar during the treatment procedure.

The *Vito App* is a tele-exercise-based mHealth application developed by Hessam et al. in 2022 that provides exercises for PwPFPS in 6 weeks. The application’s usability was evaluated and received high scores from both physical therapists and PwPFPS who used it [37]. This application has three sections: information and advice, exercise plan, and calendar.

**Fig. 2:**
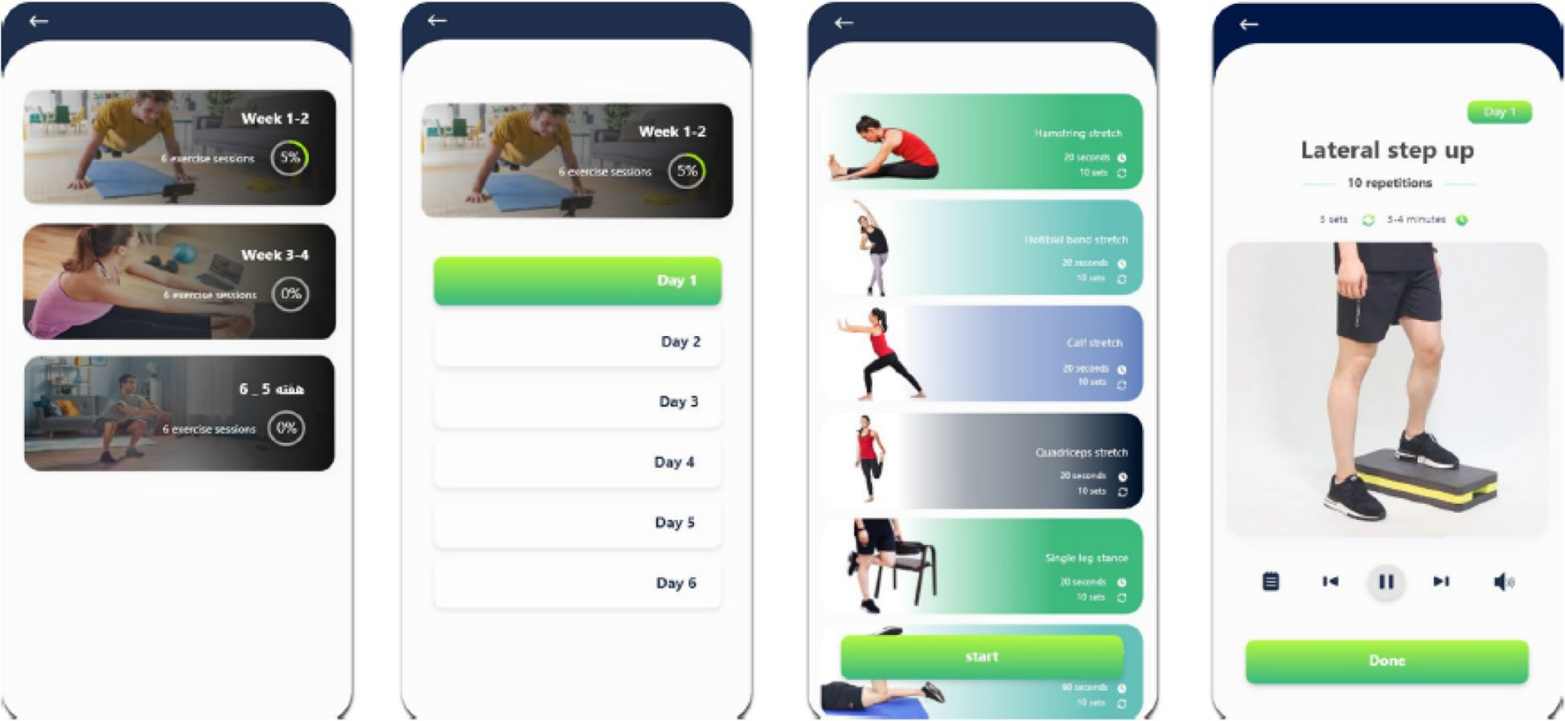
The *Vito App* application; Exercise plan. Figure adapted with permission from Hessam et al. (2022) [37]

**Fig. 3:**
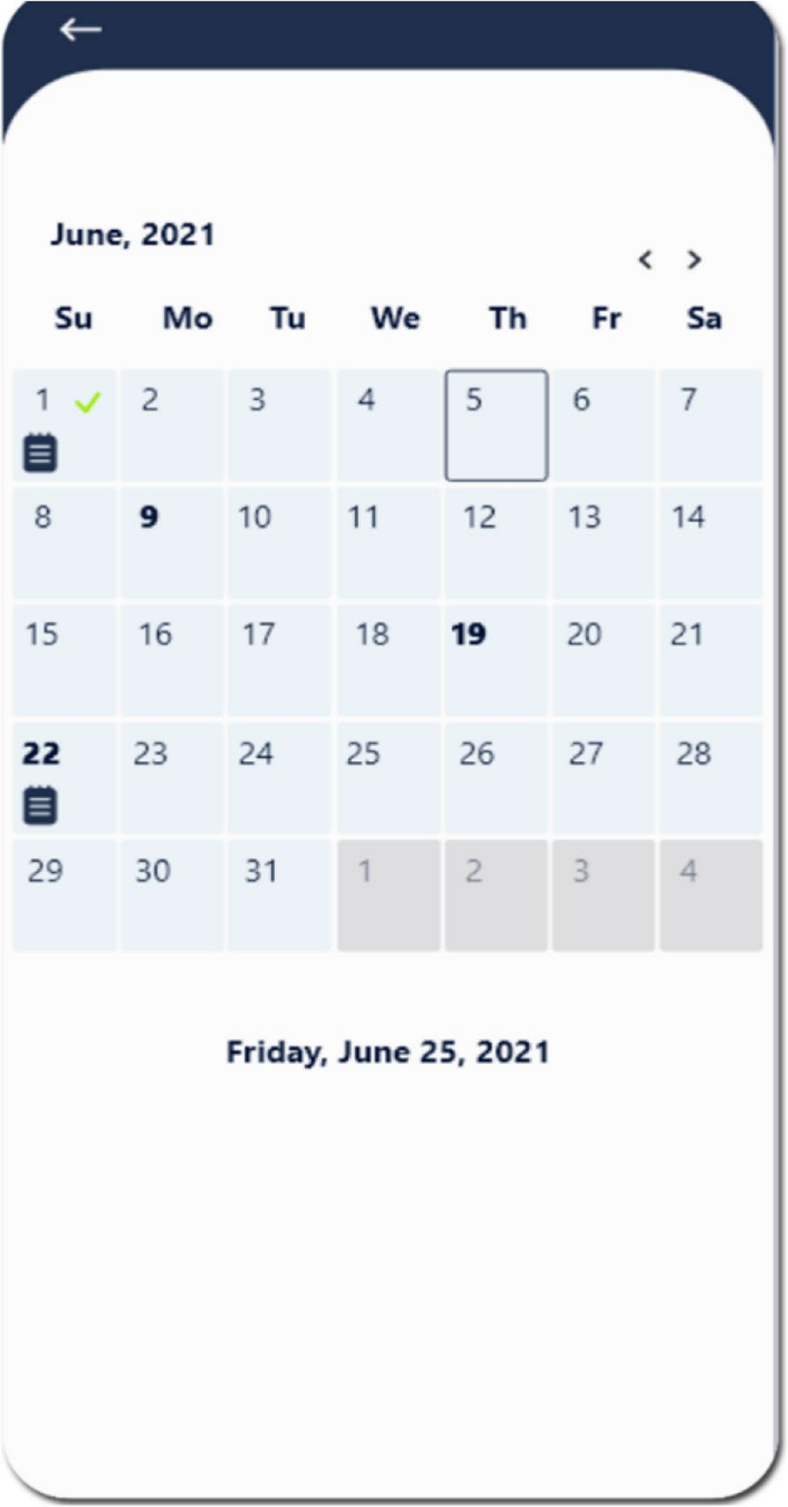
The *Vito App* application; Exercise plan calendar. Figure adapted with permission from Hessam et al. (2022) [37]

- Information and advice: This section briefly explains PFPS and its signs and symptoms, medications, home treatments, and some tips about physical health.
- Exercise plan: This section includes stretching, strengthening, balance, and functional exercise videos with detailed instructions. Patients can also send messages to the physical therapist in the application and report any problem they had with their exercise plan or application (Fig. 2)
- Calendar: The application includes a monthly calendar that displays the days on which participants exercise, their pain intensity, and their performance on the exercises (Fig. 3)

### Outcome measures

The outcome measures will be assessed in three phases: pre-test, post-test (after 6 weeks and 18 sessions), and follow-up (1 month after the final session) to evaluate the mid-term effects of the intervention. The same physical therapist (F.M.), who is blinded to the participant’s group allocation, will evaluate the outcome measures in every phase in a random order. All assessments were conducted at the Rehabilitation Research Center, AJUMS, Ahvaz, Iran.

### Primary outcome measures

The primary outcomes include self-reported pain intensity, physical function, and quality of life as follows:

- Pain intensity:
  The VAS measures self-reported pain intensity. On this scale, 0 indicates “no pain,” and 10 indicates “the worst possible pain”. Patients will be asked to report their knee pain intensity in rest and the maximum knee pain intensity they have ever experienced in the last month.
- Knee physical function will be assessed by the Persian version of the Kujala questionnaire and three functional tests, including step down, bilateral squat, and anteromedial lunge.
  Kujala or Anterior Knee Pain Scale (AKPS); consists of 13 self-reported questions about knee physical function with a score that ranged on a scale of 0 to 100, with 100 being the highest possible score and no signs of anterior knee pain. The Persian version of this scale is a reliable and valid tool with acceptable test-retest reliability (ICC = 0.96) and internal consistency (Cronbach’s *ɑ* = 0.81) in Iranian patients with PFPS [38].
  The Step Down Test is a unilateral test performed from a 20-cm high step. Participants step forward and lower the uninvolved limb to the floor so that the heel of the uninvolved limb touches the floor and then comes back to full knee extension on the step. This counts as one repetition. The number of repetitions in 30 s will be recorded. The Step Down Test has acceptable test-retest reliability (ICC = 0.94) in PwPFPS [39].
  The Bilateral Squat Test is a full weight-bearing test to assess patellofemoral joint function. Participants have to stand with their knees in full extension and feet shoulder-width apart. They are asked to go to 90° squat position and then return to the starting position. This counts as one repetition. The number of repetitions in 30 s will be recorded. The ICC level of this measure is 0.79 in PwPFPS [39].
  The anteromedial lunge test is a functional test that challenges the patellofemoral joint with valgus stress. The participant is asked to lunge forward so the front leg bends to 90°. The distance from the start line to the heel of the front leg will be measured. The participant should repeat the trial three times, and 80% of the maximum distance will be marked on the ground. The number of lunges that pass the 80% mark in 30 s will be recorded. The ICC level of this measure is 0.82 in PwPFPS [39].
- Quality of life:
  The Persian version of the Knee Injury and Osteoarthritis Outcome Score (KOOS) includes the quality of life subscale, which is a valid and reliable tool for assessing the quality of life in PwPFPS [40, 41]. KOOS is a self-reported questionnaire consisting of five categories: pain, other symptoms, function in daily living, function in sport and recreation, and knee-related quality of life. Knee related quality of life subscale comprises four questions about the effect of pain and knee discomfort on quality of life.

### Secondary outcome measures

The secondary outcome measures include psychometric features of anxiety and depression, pain catastrophizing, and kinesiophobia, and also the adherence of patients to treatment sessions.

**Fig. 4:**
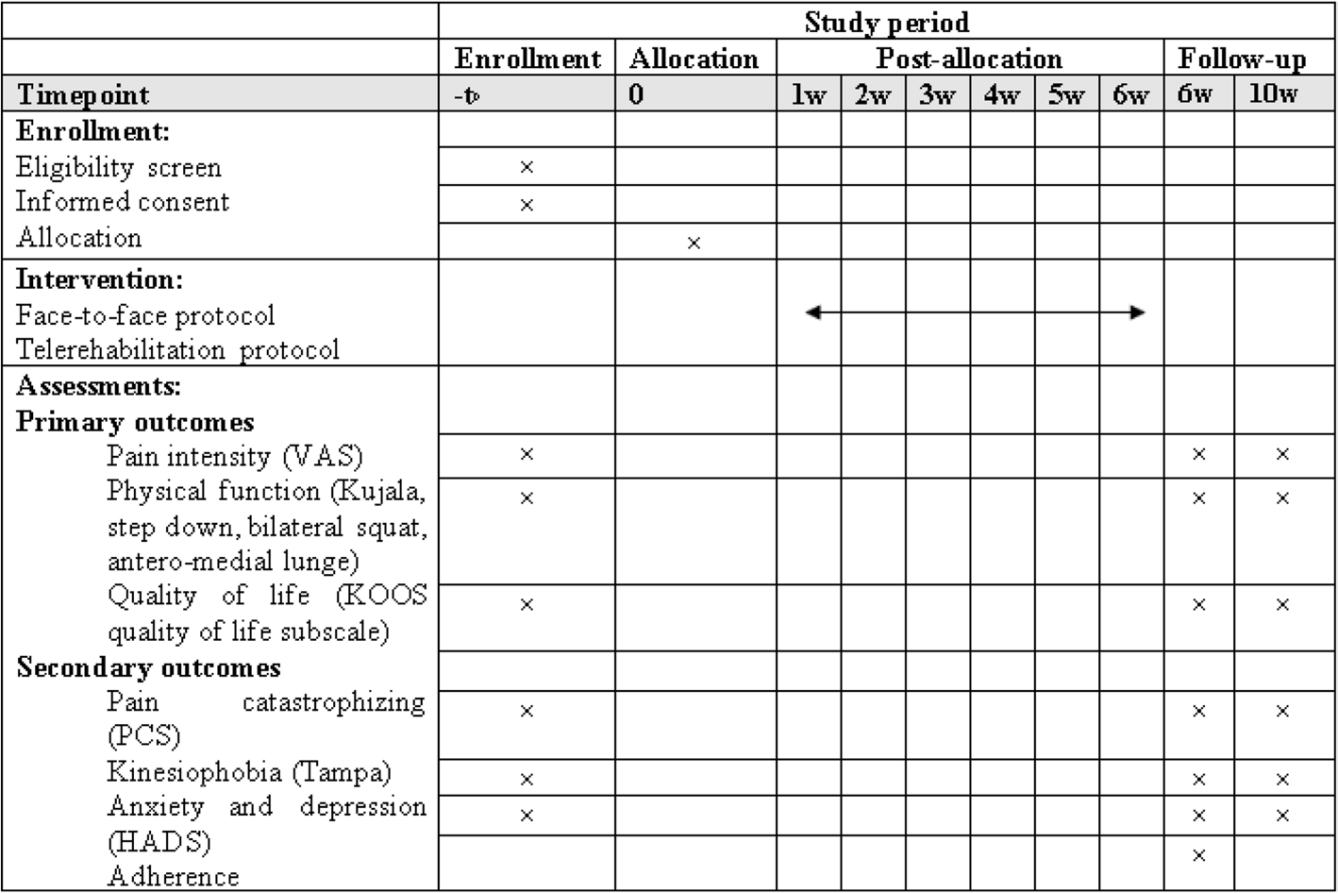
Standard protocol items: schedule of enrollment, interventions, and assessments (according to SPIRIT)

- Hospital Anxiety and Depression Scale (HADS) will evaluate anxiety and depression. This questionnaire comprises 14 items and two subscales (anxiety and depression). Each subscale consists of 7 questions. Each item is scored on a 4-point scale. The total score ranges from 0 to 21. A subscale score of more than 8 denotes anxiety or depression. The Persian version of HADS is a valid and reliable scale to evaluate anxiety and depression in Iranian anxious and depressed patients [42].
- Pain catastrophizing will be measured by the Pain Catastrophizing Scale (PCS). It has 13 items and three subscales (rumination, magnification, and helplessness). The range of total score is from 0 to 52, with higher scores indicating higher amounts of pain catastrophizing. The Persian version of PCS is valid and reliable in assessing pain catastrophizing in Iranian patients suffering from pain [43].
- Kinesiophobia will be assessed using the Tampa Scale. Tampa is a questionnaire with 17 items that evaluates fear of movement. Each item is scored on a scale of 1 to 4. The total score ranges from 17 to 68, with higher scores indicating greater fear of movement. The Persian version of the Tampa Scale has high reliability and validity for individuals with chronic pain [44, 45].
- Adherence to the treatment sessions in the telerehabilitation group will be evaluated by the calendar of the application and the sessions in which the participants will perform the exercises. In the control group, adherence will be assessed by the physical therapist recording the dates of the sessions on the participant’s treatment sheet. The time schedule of study was shown in Fig. 4.

### Statistical methods

The statistical analysis will be performed by SPSS (Statistical Package for Social Science) version 26.0. Data will be reported as follows: mean, standard deviation, 95% confidence intervals, or frequency and percentiles. Descriptive statistics will be obtained on the demographic and clinical characteristics of the participants. Repeated-measures ANOVA (Analysis of Variance) will be used to determine the effects of the group assignments (telerehabilitation vs. face-to-face), the effects of time (baseline, post-intervention, and 1-month follow-up), and the group-by-time interaction for all the outcome measures. *P*-values less than 0.05 will be considered to be statistically significant in all tests. Missing data will be handled using an intention-to-treat (ITT) analysis approach.

### Sample size

The sample size of 60 participants (30 per group) was determined using G*Power software (version 3.1.9.2) based on the input parameters of a power of 0.80, an α level of 0.05, and repeated-measures analyses of variance (ANOVAs) with within-between interaction statistical tests.

## Discussion

Telerehabilitation can have comparable results compared with face-to-face physical therapy to reduce pain and improve physical function and quality of life in patients with various musculoskeletal problems [46]. Telerehabilitation methods can also save time for both patients and physical therapists [28–32]. Since there are limited studies that have investigated the effectiveness of telerehabilitation in PwPFPS, the present study aims to evaluate the effectiveness of telerehabilitation via a smartphone application named *Vito App* in PwPFPS and to compare its efficacy on pain, physical function, quality of life, and psychological features with face-to-face physical therapy.

As long as PwPFPS are active and young adults, a smartphone application available for exercise therapy at home may be helpful for these populations. Using telerehabilitation methods can increase patients’ adherence to the treatment procedure, and they can actively control their condition and the rehabilitation process [15, 17].

The *Vito App* is an innovative tool for exercise therapy and learning beneficial tips about PFPS. The physical therapist can oversee the patient’s progress, and therapist-patient communication will be enhanced. The exercise therapy program in this study will be based on the guidelines of physical therapy in PFPS, and it consists of stretching, strengthening, balance, and functional exercises. Exercise therapy is the most effective treatment for PwPFPS and can lead to pain reduction and physical function improvements [11–14]. This study takes an important step in treating PwPFPS and reducing the risk of developing patellofemoral osteoarthritis later in life.

Based on our hypothesis, participants in the telerehabilitation group and face-to-face physical therapy group will have the same improvements in clinical and psychological outcomes. If our results indicate that telerehabilitation is not inferior to face-to-face physical therapy, it can be an appropriate alternative for PwPFPS, due to its advantages over face-to-face physical therapy, such as the active role of the patients in managing their condition and the fact that there will be no need to refer directly to the physical therapist. PwPFPS living in rural or remote areas or those who do not have enough time to refer to physical therapy clinics can benefit from telerehabilitation methods. PwPFPS can also continue their exercise program as long as they want to use the *Vito App*, and the physical therapists can always have accessibility to their patient’s condition, and they can monitor their progress.

The present study has some limitations, such as the impossibility of blinding the participants and interventional physical therapists due to the nature of the intervention. Another limitation is the slow internet connection in Iran and application bugs that may hinder participants in the telerehabilitation group from using the application. Additionally, using a smartphone application may exclude participants who do not have access to the internet or smartphones.

## Trial status

The trial was registered on 29 October 2022, under the registration IRCT20201112049361N1. Patient recruitment and data collection are currently ongoing and will continue until the required number of participants is achieved. Recruitment was initiated on 1 November 2022 (study protocol version 1, dated July 2022), and is expected to be completed by the end of November 2023. The results of this trial will be submitted to a peer-reviewed journal after the recruitment completion. The research team will have a meeting every 3 months to investigate possible problems during the research (intervention, tests, and follow-ups). In addition, a supervisor (who is not a member of the research team) has been determined by the ethics committee of AJUMS to monitor the correct conduct of the research.

### Supplementary Information

**Additional file 1.** SPIRIT checklist.

## Data Availability

The datasets used and/or analyzed during the current study will be available upon reasonable. request to the corresponding author after the main publication of them.

## Abbreviations

PFPS: Patellofemoral pain syndrome
PwPFPS: People with PFPS
VAS: Visual analog scale
KOOS: Knee Injury and Osteoarthritis Outcome Score
HADS: Hospital Anxiety and Depression
PCS: Pain Catastrophizing Scale
IRCT: Iranian Registry of Clinical Trials
mHealth: Mobile health
AJUMS: Ahvaz Jundishapur University of Medical Sciences
APTA: American Physical Therapy Association
AKPS: Anterior Knee Pain Scale
SPSS: Statistical Package for Social Science
ANOVA: Analysis of variance
ITT: Intention-to-treat
